# Detection of adverse event signals associated with key immunosuppressants for autoimmune hepatitis: insights from the food and drug administration adverse event reporting system database

**DOI:** 10.3389/fphar.2026.1889345

**Published:** 2026-09-24

**Authors:** Luyuan Ma, Yize Fan, Ruolan Gu, Ziyue Li, Yuexia Liu, Kaixuan Zhang, Xiaochao Gao, Caiyan Zhao

**Affiliations:** 1 Department of Infectious Diseases, Hebei Medical University Third Hospital, Shijiazhuang, China; 2 Department of Burn and Plastic Surgery Department, Hebei Medical University Third Hospital, Shijiazhuang, China; 3 Department of Allergy Department, Hebei Medical University First Hospital, Shijiazhuang, China

**Keywords:** autoimmune hepatitis, adverse drug events, glucocorticoids, azathioprine, mycophenolate mofetil, FAERS, pharmacovigilance

## Abstract

**Background:**

Autoimmune hepatitis (AIH) requires long-term immunosuppressive therapy, yet the comparative safety profiles of key drugs remain incompletely characterized. The objective of this study was to conduct a systematic evaluation of the adverse drug event (ADE) signals associated with glucocorticoids, specifically dexamethasone, methylprednisolone, and prednisolone, in addition to azathioprine and mycophenolate mofetil, in the treatment of AIH.

**Methods:**

Disproportionality analysis was performed on ADE reports from the Food and Drug Administration (FDA) Adverse Event Reporting System (FAERS) database, covering the most recent data available from the first quarter of 2004 through the fourth quarter of 2024. Reporting odds ratios (RORs), proportional reporting ratios (PRRs), and Bayesian confidence propagation neural network metrics were calculated to identify significant ADE signals. Stratified analyses examined demographic and reporting factors influencing ADE patterns.

**Results:**

Among 720 reports, females accounted for a higher proportion of ADE reports (62.9%). Infections and gastrointestinal disorders were predominant signals across therapies. Glucocorticoid monotherapy showed strong SOC signals for infections, endocrine disorders, and metabolism and nutrition disorders, with opportunistic infections at the PT level. Azathioprine monotherapy exhibited gastrointestinal and neoplasm signals, alongside drug intolerance. Glucocorticoid-azathioprine showed gastrointestinal, infection, and drug intolerance signals; glucocorticoid-mycophenolate mofetil linked to infections. Mycophenolate mofetil monotherapy exhibited medication management failures. Stratified analyses revealed higher ADE reporting frequencies in females, patients ≥65 years, and differential reporting patterns by occupation.

**Conclusion:**

This study identified distinct ADE profiles for AIH therapies. These findings underscore the need for personalized monitoring strategies to mitigate treatment-specific ADEs in AIH management.

## 1 Introduction

Autoimmune hepatitis is a liver inflammatory disorder of unknown etiology that can advance to cirrhosis and end-stage liver failure if not promptly diagnosed and treated. Clinically, it often presents as acute hepatitis, which can be severe, but may also manifest insidiously or remain asymptomatic (Muratori et al., 2023). This condition can affect individuals of all ages, with a higher prevalence in females, and both its incidence and prevalence appear to be rising globally (Lamba et al., 2021). The pathogenesis involves an aberrant immune response against liver autoantigens, resulting in self-sustaining hepatic inflammation. (Shiffman, 2024). A rapid therapeutic response to corticosteroids and other immunomodulatory agents is typically observed and serves to confirm the diagnosis. The primary objective of treatment is to achieve and maintain long-term remission of hepatic inflammation. Treatment may also reduce or even reverse liver fibrosis, thereby preventing progression to advanced cirrhosis and its associated complications. However, most patients require ongoing management due to the high risk of disease relapse upon treatment discontinuation.

Currently, the standard first-line treatment for AIH involves the use of immunosuppressive agents. A combination of corticosteroids, such as prednisolone, and azathioprine (AZA) constitutes a well-established induction and maintenance therapy designed to achieve and sustain long-term remission of hepatic inflammation and potentially reverse liver fibrosis (Galaski et al., 2021; Reau et al., 2024; Dalekos et al., 2025). While this treatment regimen demonstrates high efficacy and aids in diagnostic confirmation, the majority of patients require long-term or lifelong maintenance therapy due to the elevated risk of disease recurrence following discontinuation (Komori, 2021; Schregel et al., 2024). Nevertheless, the adverse effects associated with prolonged use render the clinical management of AIH particularly challenging. In particular, the extended use of glucocorticoids, including prednisolone, dexamethasone, and methylprednisolone, in conjunction with azathioprine, is associated with significant adverse effects. Corticosteroids are linked to a spectrum of side effects, such as hypertension, peptic ulcers, hyperglycemia, electrolyte imbalances, and osteoporosis. The poor water solubility of certain glucocorticoid formulations, such as dexamethasone, may further contribute to systemic adverse effects (van den Brand et al., 2019). AZA is associated with tolerability concerns, as some patients experience adverse reactions such as bone marrow suppression, hepatotoxicity, and gastrointestinal disturbances, which preclude continued use (Şimşek et al., 2024). Given the limitations of conventional therapies, it is imperative to identify safer and more efficacious alternatives. Several alternatives to azathioprine, including mycophenolate mofetil, tacrolimus, cyclosporine, sirolimus, and rituximab, have been assessed in patients with inadequate response to standard treatment. Mycophenolate mofetil (MMF), a potent and selective immunosuppressive agent that inhibits lymphocyte proliferation, is the primary second-line treatment option supported by substantial evidence. More recently, it has emerged as a viable alternative first-line therapy (Goel and Kwo, 2024; Şimşek et al., 2024; Ali et al., 2025). Studies indicate that, in comparison to AZA, the combination of MMF and prednisolone results in a higher rate of biochemical remission and a lower incidence of adverse events in patients newly diagnosed with AIH(Santiago et al., 2019; Dalekos et al., 2022; Ali et al., 2025). Compared with MMF, AZA is associated with a higher incidence of adverse events, which are also more severe. However, both AZA and MMF can cause fatigue, infections, and gastrointestinal disorders, as well as hematologic abnormalities, including reduced white blood cell, neutrophil, and platelet counts, and elevated serum creatinine levels. Thus, a thorough evaluation of the long-term safety of these commonly used drugs is needed, focusing on the characteristics and reporting patterns of drug-related adverse events in real-world AIH populations.

The Food and Drug Administration (FDA) Adverse Event Reporting System (FAERS) is a database maintained by the United States Food and Drug Administration. It collects spontaneous reports globally from consumers, healthcare professionals, and pharmaceutical companies for post-market drug safety monitoring. Current clinical management of AIH typically employs a staged treatment strategy (Mack et al., 2020). However, the spontaneous reporting nature of the FAERS database limits the ability to differentiate safety signals between induction and maintenance phases. This study aims to evaluate the overall safety signals of key drugs throughout the treatment process, providing a comprehensive signal overview for clinical research. Additionally, although monotherapy diverges from practice guidelines (Li et al., 2025), it is still used in cases where other immunosuppressants are intolerable or contraindicated. Therefore, this study includes glucocorticoids to assess their safety signals and independent reporting associations in AIH therapy. We analyzed ADEs associated with five key immunosuppressive drugs commonly used for AIH: dexamethasone, methylprednisolone, prednisolone, azathioprine, and mycophenolate mofetil. Using FAERS data from Q1 2004 to Q4 2024, this study applied disproportionality analysis and data mining techniques to identify and quantify reported ADEs. The goal was to provide safety insights to aid clinicians in optimizing immunosuppressive therapy selection and monitoring strategies for AIH, ultimately improving treatment safety and effectiveness.

## 2 Materials and methods

### 2.1 Data source

This study utilized data from the United States Food and Drug Administration Adverse Event Reporting System (FAERS) database, a publicly accessible, spontaneous reporting system serving as a post-marketing repository for adverse drug event (ADE) reports, product quality complaints, and medication error reports associated with FDA-approved therapies. Data spanning from the first quarter of 2004 to the fourth quarter of 2024 (2004 Q1 - 2024 Q4) were extracted, ensuring the analysis reflects contemporary prescribing and reporting patterns. Reports involving the drugs were identified using the corresponding drug names as keywords. Subsequently, reports where “AIH” was listed as an indication were specifically extracted from the raw data. Patient information, including DEMO, DRUG, REAC, OUTC, RPSR, THER, and INDI data, was retrieved for these reports. Reports were rigorously screened according to predefined exclusion criteria: 1) Reports officially deleted by the FDA, duplicate reports, or reports missing a valid ID for AE reports related to AIH treatment with the target drugs were excluded; 2) Reports with inaccurate or missing information regarding gender, age, weight, or reporting country were excluded. All included data underwent standardized processing to ensure consistency. The detailed sample screening process and the distribution of reports across the five treatment regimens are presented in Supplementary Figure S1 and Supplementary Table S1, respectively.

### 2.2 Definition of treatment regimens

Each adverse event report records one primary suspect drug, and all reports are accordingly assigned to the corresponding monotherapy group. Therefore, the glucocorticoid monotherapy, azathioprine monotherapy, and mycophenolate mofetil monotherapy groups are completely mutually exclusive, and the sum of the number of reports in these three groups equals the total number of reports included in the analysis. Combination therapy groups were determined based on the actual medications used in the reports; any report in which glucocorticoids were concurrently used with azathioprine or with mycophenolate mofetil during the treatment course was classified into the respective combination group. In addition, the “other combination regimens” category included reports involving three or more of the target immunosuppressants. Due to the limited sample size and high heterogeneity of drug combinations in this group, it was included only in the temporal trend description and was not subjected to disproportionality analysis for signal detection.

### 2.3 Descriptive analysis of baseline characteristics

Demographic and clinical characteristics of patients treated with the target drugs for AIH were summarized. Continuous non-normally distributed variables (e.g., age, weight) were expressed as median (interquartile range, IQR), while categorical variables (e.g., gender) were described as frequencies and percentages (%). Temporal trends in AE reporting for AIH treatment from 2004 Q1 to 2024 Q4 were visualized using the R package ggplot2 (v3.4.1) (Wang et al., 2022a).

### 2.4 Signal detection

ADEs were coded using the Medical Dictionary for Regulatory Activities (MedDRA), with preferred terms (PTs) describing specific medical concepts and system organ classes (SOCs) categorizing events by etiology, site, or purpose. Given the high rate of missing and non-uniform dosage data in FAERS, dose-based stratification was not feasible. Therefore, signal detection relied solely on the presence (exposure) of the target drugs, not on specific doses. All disproportionality analyses used all reports in the entire FAERS database during the study period as the background control population, rather than being limited to the subset of reports for which AIH was the indication. For each target drug, the target group consisted of reports containing that drug and the target ADE; all other reports (those not containing the drug, or those containing the drug but not the target ADE) were included in the background control calculations. Three established disproportionality analysis methods were employed for signal detection, including the Reporting odds ratio (ROR), the proportional reporting ratio (PRR), and the Bayesian confidence propagation neural network (BCPNN). The ROR and PRR are frequentist methods that calculate the ratio of observed-to-expected reporting frequencies, offering high sensitivity for signal generation. In contrast, the BCPNN is a Bayesian approach that calculates the Information Component (IC), providing a more stable estimation for rare events by incorporating prior probabilities, thereby enhancing specificity. Their combined use aimed to complement each other and enhance the robustness of signal identification amidst the inherent variability of spontaneous reporting data. For ROR, a signal was considered significant if the number of co-reported cases for a specific drug-AE pair (a) was ≥3 and the lower limit of the 95% confidence interval (CI) for the ROR was >1. For PRR, a signal was deemed significant if the number of co-reported cases (a) was ≥3, the PRR value was ≥2, and the Chi-square (χ^2^) value was ≥4. The BCPNN method focused on the IC. A signal was considered significant if the IC lower 95% credibility interval (IC025) was >0. An ADE signal was ultimately defined as valid if it met the criteria for significance in any one of the three methods (ROR, PRR, or BCPNN). The strength of the signal, indicating the potential association strength between the target drug and the specific AE, was interpreted based on the magnitude of the signal metric. The two-by-two contingency table and detailed formulas for these methods of disproportionality analysis and the positive signal thresholds are provided in Supplementary Table S2.

### 2.5 Stratified analysis

To further investigate the impact of different stratification schemes on ADEs associated with the target drugs in AIH treatment, stratified analyses were conducted. Subgroups were defined based on sex (female and male), age (<65 and ≥65 years), and reporter occupation (consumer/lay-witness versus health-care professionals).

### 2.6 Statistical analysis

All statistical analyses and data processing were performed using R software (v 4.2.3).

## 3 Results

### 3.1 Demographic and reporting characteristics of ADEs associated with AIH treatment

Analysis of ADEs associated with the target drugs used for AIH treatment revealed distinct demographic and reporting patterns. Female patients accounted for a significantly higher proportion of reported ADEs (453 cases, 62.9%) compared to male patients (177 cases, 24.6%). Among adults, individuals with a body weight ranging from 50 to 100 kg constituted a relatively higher proportion of the reported cases. Geographically, the majority of ADE reports originated from the United States (213 cases, 29.6%). Regarding the source of reporting, submissions by physicians (MD) accounted for the highest proportion of cases (212 cases, 29.4%) (Supplementary Table S3).

### 3.2 Temporal trends in ADE reporting

The temporal evolution of ADE reporting associated with AIH treatment using the target drugs was assessed over the 20 years from 2004 Q1 to 2024 Q4. Between 2005 and 2009, the annual number of reported ADEs displayed minor fluctuations accompanied by a gradual upward trend. A decline in reported ADE occurrences was observed during the period 2009 to 2011. Subsequently, from 2011 to 2018, a more pronounced increase in reporting frequency was evident. This increase culminated in distinct peaks for different therapeutic strategies: ADEs associated with glucocorticoid monotherapy reached their highest point in 2018, while ADEs linked to mycophenolate mofetil peaked in 2019. Commencing in 2020, the annual number of reported ADEs exhibited a pattern characterized by minor fluctuations (Figure 1). The above trend reflects annual changes in the number of FAERS reports. Between 2004 and 2024, systemic factors, such as the widespread adoption of electronic reporting systems, the expansion of mandatory reporting requirements, and the increased global participation in pharmacovigilance, may have influenced the temporal distribution of these reports; therefore, this trend should not be directly equated with dynamic changes in the actual risks associated with AIH treatment.

**FIGURE 1:**
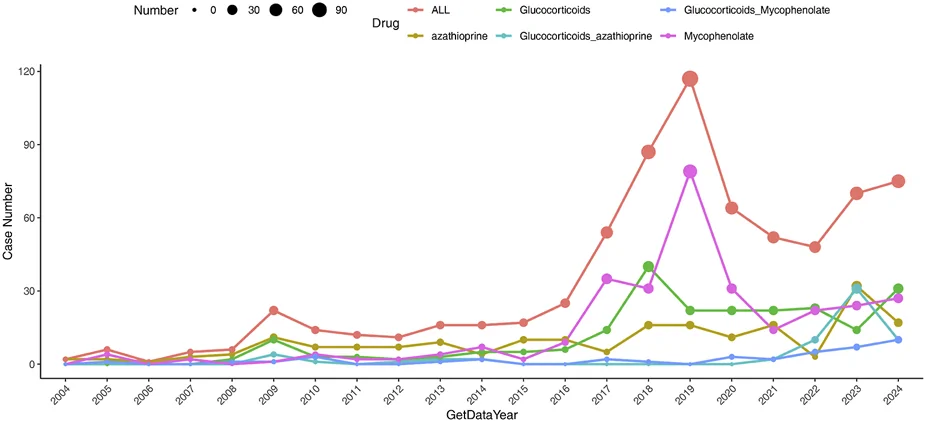
Temporal trends in adverse drug event (ADE) reporting for autoimmune hepatitis (AIH) treatment strategies (2004 Q1–2024 Q4). Red represents the use of azathioprine alone, turquoise represents the combined use of glucocorticoids and azathioprine, green represents the use of glucocorticoids alone, blue represents the combined use of glucocorticoids and mycophenolate mofetil, purple represents the use of mycophenolate mofetil alone, and yellow-green represents other combination regimens (including ≥3 immunosuppressants).

### 3.3 Overall ADE signals across all therapies at the SOC and PT level

The analysis identified significant ADE signals associated with five therapeutic strategies for AIH at both SOC and PT levels. At the SOC level, the strongest signal was for neoplasms (77 cases; ROR = 1.86, 95% CI: 1.37–2.53; ROR, BCPNN), followed by infections and infestations (337 cases; ROR = 1.52, 95% CI: 1.31–1.76; ROR, BCPNN) and gastrointestinal disorders (227 cases; ROR = 1.24, 95% CI: 1.04–1.47) (Supplementary Table S4; Figure 2A).

**FIGURE 2:**
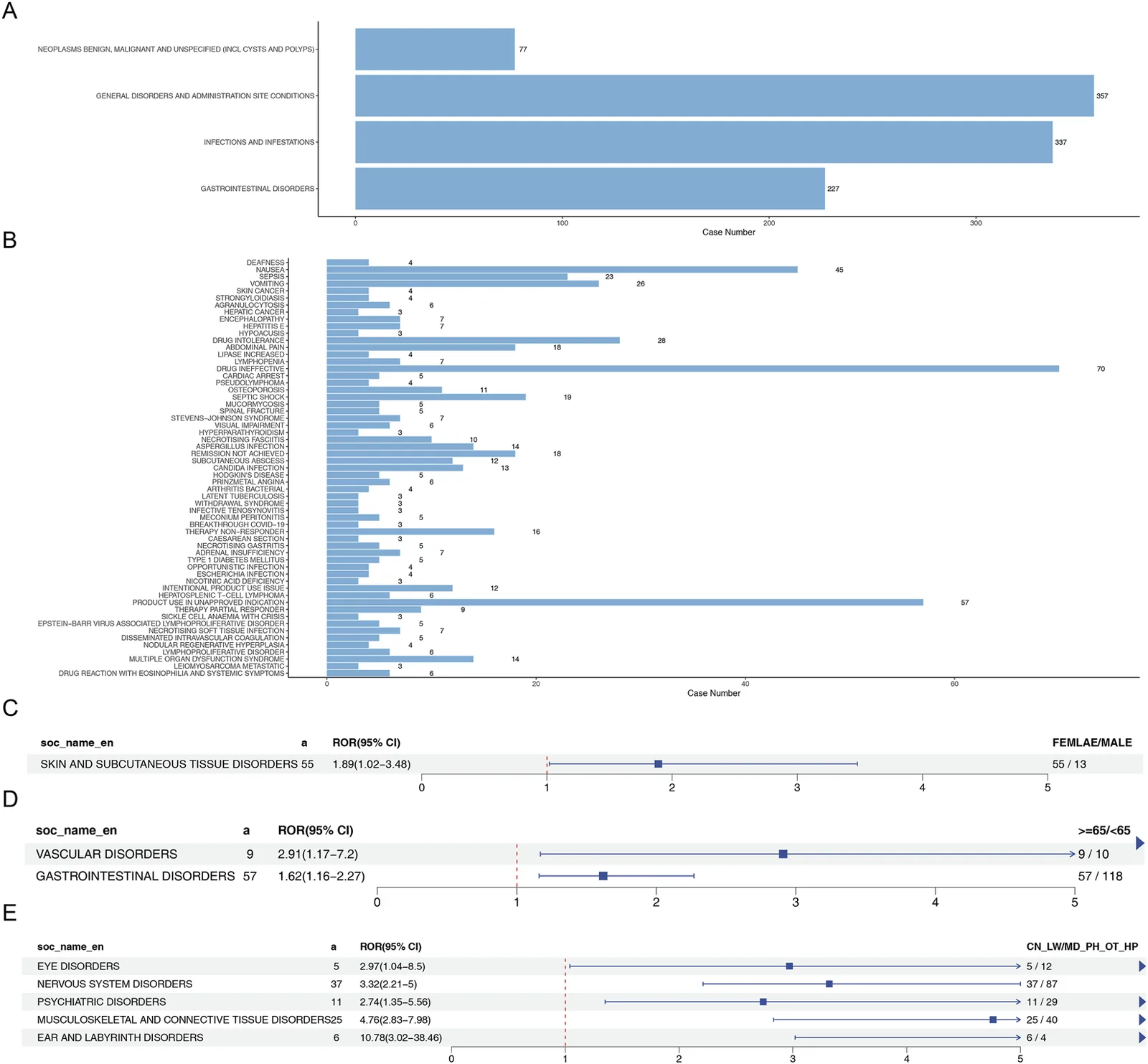
Disproportionality analysis of ADE signals for AIH therapies at system organ class (SOC) and preferred term (PT) levels. **(A)** Analysis of SOC level, with the number of adverse event reports on the abscissa and adverse events of SOC level on the ordinate. **(B)** Case map of ADEs of five drugs for treating AIH at the PT level in Food and Drug Administration (FDA) Adverse Event Reporting System (FAERS). **(C)** Gender-stratified analysis of ADEs reported in association with the five target drugs. **(D)** Age-stratified analysis of ADEs reported in association with the five target drugs. **(E)** Occupation-stratified analysis of ADEs reported in association with the five target drugs.

At the PT level, among PTs with at least 10 reported cases, subcutaneous abscess (12 cases; ROR = 13.04, 95% CI: 2.91–58.29; ROR, PRR, BCPNN) showed the largest ROR, followed by drug intolerance (28 cases; ROR = 12.25, 95% CI: 4.72–31.76; ROR, PRR, BCPNN), aspergillus infection (14 cases; ROR = 4.34, 95% CI: 1.75–10.78; ROR, PRR), abdominal pain (18 cases; ROR = 3.91, 95% CI: 1.80–8.49; ROR, PRR, BCPNN), and *candida* infection (13 cases; ROR = 2.82, 95% CI: 1.24–6.44; ROR, PRR) (Supplementary Table S5; Figure 2B). Although infections and gastrointestinal disorders were frequent at the SOC level, the PT-level signals were dominated by severe infections (subcutaneous abscess, aspergillus, and *candida*) and drug intolerance, suggesting that clinical monitoring should prioritize invasive fungal infections and treatment tolerability.

### 3.4 Stratified analysis of ADEs

To evaluate the impact of gender, age, and reporter occupation on ADE reporting patterns in AIH treatment. Gender-based stratification revealed significant disparities in ADE reporting patterns. At the SOC level, skin and subcutaneous tissue disorders exhibited pronounced female predominance (Female/Male = 55/13; ROR = 1.89, 95%CI = 1.02–3.48) (Figure 2C). Age stratification revealed a higher frequency of ADEs among patients aged ≥65 years. At the SOC level, significant associations were observed for both vascular disorders (≥65/<65 = 9/10; ROR = 2.91, 95% CI: 1.17–7.20) and gastrointestinal disorders (≥65/<65 = 57/118; ROR = 1.62, 95% CI: 1.16–2.27) (Figure 2D). Stratification by reporter occupation revealed a higher reporting frequency among healthcare professionals (MD_PH_OT_HP) compared to consumers/lay witnesses (CN_LW). At the SOC level, significant inter-occupational reporting differences were observed for nervous system disorders (CN_LW/MD_PH_OT_HP = 37/87; ROR = 3.32, 95% CI: 2.21–5.00), musculoskeletal and connective tissue disorders (25/40; ROR = 4.76, 95% CI: 2.83–7.98), and psychiatric disorders (11/29; ROR = 2.74, 95% CI: 1.35–5.56) (Figure 2E). These findings underscore the importance of demographic and occupational factors in ADE surveillance, highlighting the need for age-specific monitoring protocols and consideration of potential reporting biases. The differential reporting patterns between professional and non-professional groups may reflect variations in symptom recognition thresholds or clinical significance assessment.

### 3.5 Identification of significant ADE signals for glucocorticoid monotherapy in AIH

We systematically identified significant ADE signals associated with AIH therapy. At the SOC level, strongest signal was for infections and infestations (176 cases; ROR = 2.90, 95% CI: 2.40–3.50; ROR, PRR, BCPNN), followed by metabolism and nutrition disorders (30 cases; ROR = 1.91, 95% CI: 1.28–2.86; ROR and BCPNN) and endocrine disorders (16 cases; ROR = 2.76, 95% CI: 1.57–4.85; ROR, PRR, and BCPNN) (Supplementary Table S6; Figure 3A).

**FIGURE 3:**
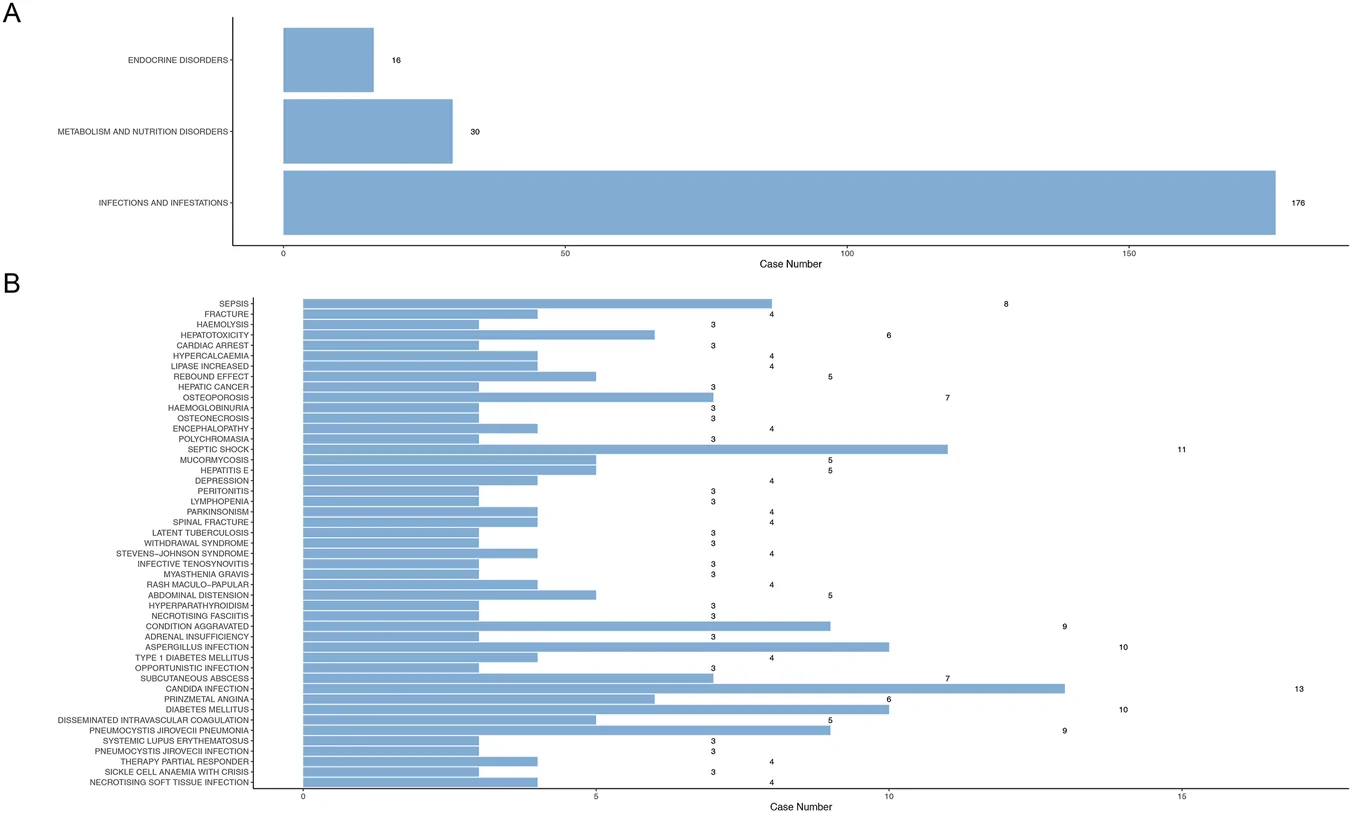
Significant ADE signals for glucocorticoid monotherapy in AIH. **(A)** Case map of ADEs of AIH treated with glucocorticoids alone in FAERS at the PT level. **(B)** SOC level analysis, with the abscissa representing the number of adverse event reports and the ordinate representing the adverse events at the SOC level.

At the PT level, robust signals were identified for opportunistic infections including *candida* infection (13 cases; ROR = 12.14, 95%CI = 5.30–27.79; ROR, PRR, and BCPNN), aspergillus infection (10 cases; ROR = 8.45, 95%CI = 3.58–19.97; ROR, PRR, and BCPNN), and pneumocystis jirovecii pneumonia (9 cases; ROR = 5.97, 95%CI = 2.57–13.83; ROR, PRR, and BCPNN). Metabolic complications were represented by diabetes mellitus (10 cases; ROR = 4.89, 95%CI = 2.26–10.55; ROR, PRR, BCPNN). Severe systemic events included septic shock (11 cases; ROR = 3.40, 95%CI = 1.70–6.82; ROR, PRR, BCPNN), subcutaneous abscess (7 cases; ROR = 9.26, 95%CI = 3.24–26.49; ROR, PRR, BCPNN), and the rare cardiovascular event prinzmetal angina (6 cases; ROR = 55.56, 95%CI = 6.68–462.14; ROR, PRR, BCPNN) (Supplementary Table S7; Figure 3B). It should be noted that some of these signals are based on a small number of cases, and their clinical significance requires further data for evaluation. Overall, the above signals suggest that the safety profile of glucocorticoid monotherapy warrants particular attention regarding infectious events and metabolic disorders.

The above signal analysis for glucocorticoid monotherapy in AIH aggregates reports from all dosage levels; the lack of dose stratification prevents differentiation of reporting patterns between induction and maintenance therapy phases. As glucocorticoid monotherapy regimen is not guideline-recommended for long-term use, these signals are likely derived from off-protocol clinical scenarios rather than standard treatment settings.

### 3.6 Identification of significant ADE signals for azathioprine monotherapy in AIH

Azathioprine monotherapy yielded a signal profile dominated by gastrointestinal disorders, dermatological conditions, and neoplasm-related events. At the SOC level, the strongest association, as indicated by the highest ROR value, was observed for neoplasms (43 cases; ROR = 3.50, 95%CI = 2.45–4.99; ROR, PRR, BCPNN). Statistically significant signals were also identified for gastrointestinal disorders (107 cases; ROR = 2.16, 95%CI = 1.73–2.71; ROR, BCPNN), and skin and subcutaneous tissue disorders (37 cases; ROR = 1.73, 95%CI = 1.21–2.48; ROR, BCPNN) (Supplementary Table S8; Figure 4A).

**FIGURE 4:**
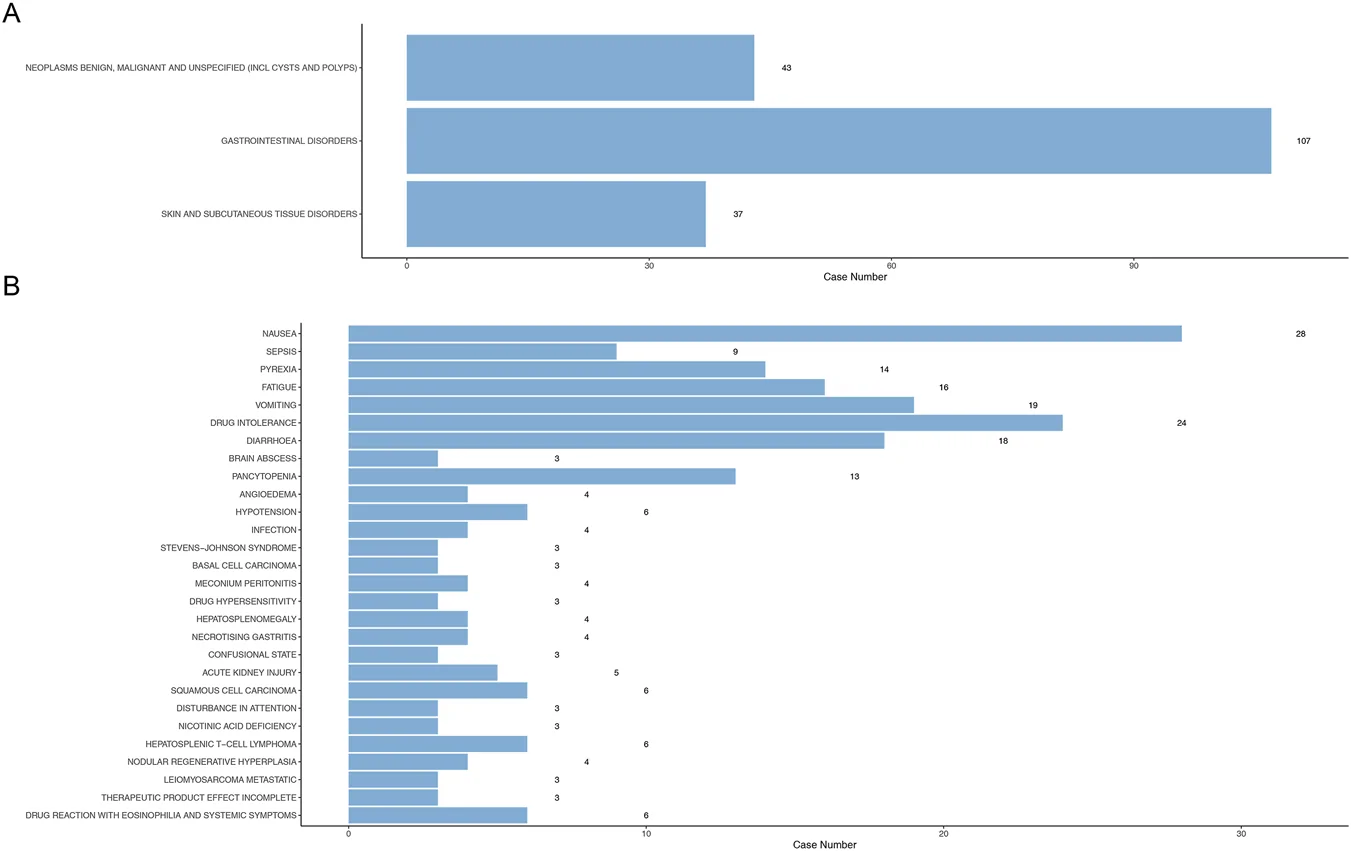
Significant ADE signals for azathioprine monotherapy in AIH. **(A)** Analysis of SOC level, with the number of adverse event reports on the horizontal axis and adverse events of SOC level on the vertical axis. **(B)** Case map of ADE of azathioprine in the treatment of AIH at the PT level in FAERS.

At the PT level, drug intolerance demonstrated the most robust signal (24 cases; ROR = 26.79, 95%CI = 12.40–57.87; ROR, PRR, BCPNN). Significant gastrointestinal intolerance signals included nausea (28 cases; ROR = 4.32, 95%CI = 2.75–6.77; ROR, PRR, BCPNN) and vomiting (19 cases; ROR = 5.24, 95%CI = 2.99–9.19; ROR, PRR, BCPNN). Additional significant PTs included diarrhoea, fatigue, and pancytopenia (Supplementary Table S9; Figure 4B). These findings highlight gastrointestinal intolerance, dermatological hypersensitivity, drug hypersensitivity and neoplasm-related reporting signals as prominent safety signals, underscoring the need for careful patient selection and long-term monitoring in AIH patients receiving azathioprine.

### 3.7 Identification of significant ADE signals for glucocorticoid-azathioprine combination therapy in AIH

The combination of glucocorticoids and azathioprine yielded significant ADE signals upon systematic identification. At the SOC level, two significant signals were detected: infections and infestations (62 cases; ROR = 2.37, 95% CI: 1.77–3.18; ROR, PRR, BCPNN) and gastrointestinal disorders (45 cases; ROR = 2.18, 95% CI: 1.57–3.04; ROR, BCPNN) (Supplementary Table S10; Figure 5A).

**FIGURE 5:**
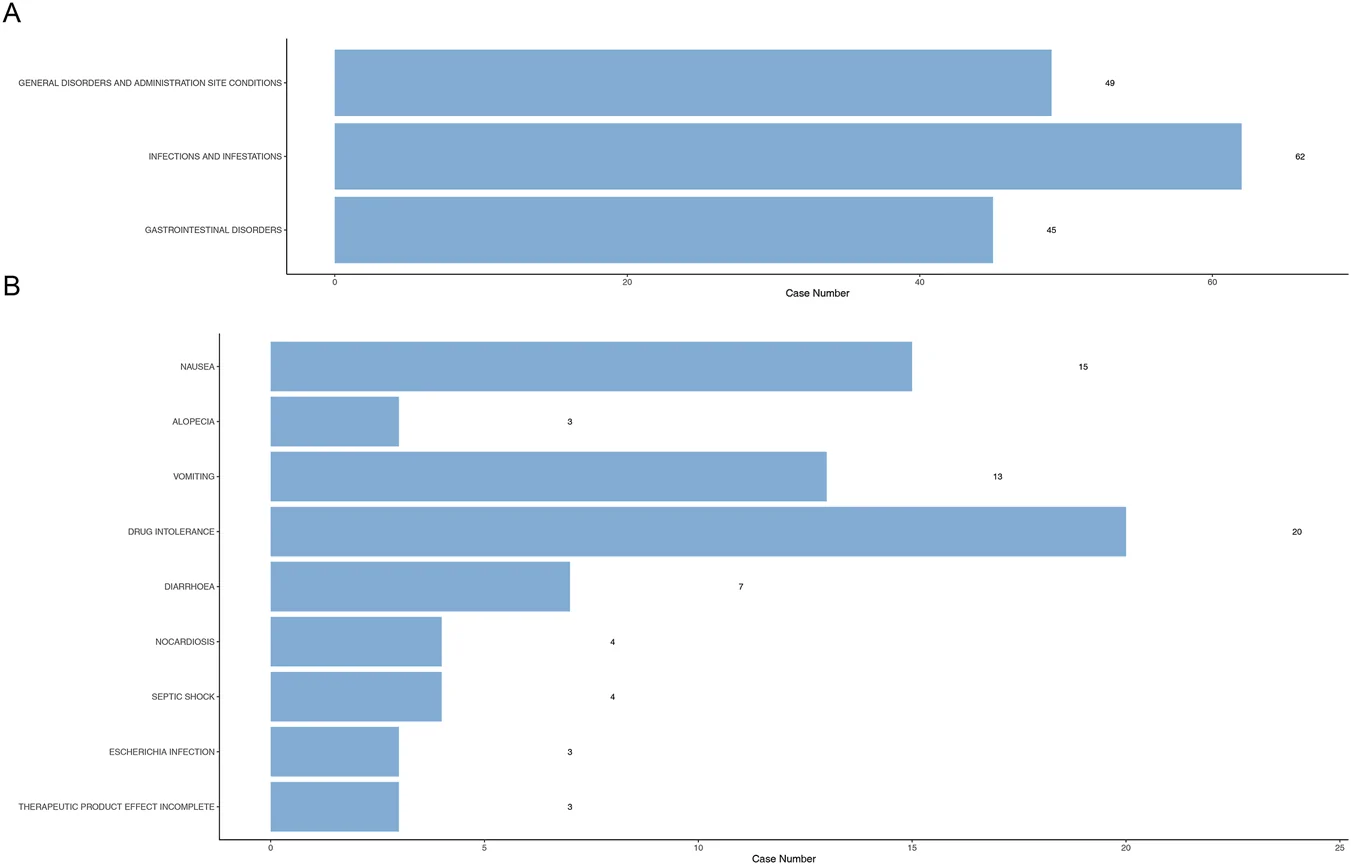
Significant ADE signals for glucocorticoid-azathioprine combination therapy in AIH. **(A)** SOC level analysis, with the horizontal axis representing the number of adverse event reports and the vertical axis representing adverse events at the SOC level. **(B)** Case map of ADEs in the treatment of AIH with glucocorticoids combined with azathioprine in FAERS at the PT level.

At the PT level, drug intolerance presented the most robust signal (20 cases; ROR = 42.9, 95%CI = 21.1–87.24; ROR, PRR, BCPNN), highlighting a critical safety issue related to treatment tolerability. Other notable PT-level signals included nausea (15 cases; ROR = 5.21, 95%CI = 2.95–9.18; ROR, PRR, BCPNN), and vomiting (13 cases; ROR = 8.36, 95%CI = 4.43–15.77; ROR, PRR, BCPNN) (Supplementary Table S11; Figure 5B). These findings underscore prominent gastrointestinal, with drug intolerance representing a critical tolerability signal. Vigilant monitoring for intolerance-related signals is warranted during combination therapy.

### 3.8 ADE signals of mycophenolate mofetil monotherapy in AIH

Mycophenolate mofetil monotherapy was systematically analyzed for significant ADE signals. At the SOC level, strongest signal was for injury, poisoning, and procedural complications (169 cases; ROR = 2.06, 95%CI = 1.71–2.48; ROR, BCPNN), followed by general disorders and administration site conditions (177 cases; ROR = 1.61, 95%CI = 1.34–1.93; ROR, BCPNN) (Supplementary Table S12; Figure 6A).

**FIGURE 6:**
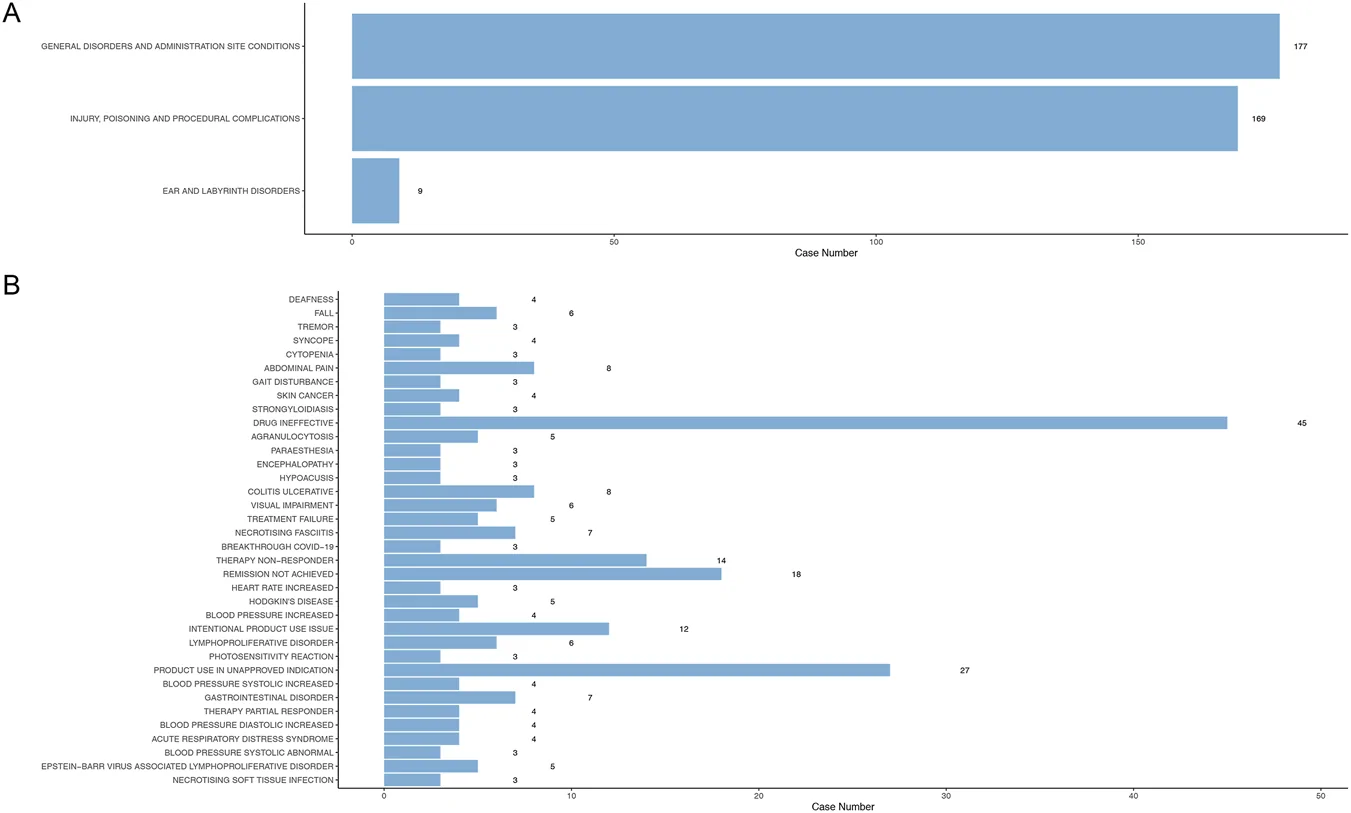
Significant ADE signals for mycophenolate mofetil monotherapy in AIH. **(A)** Analysis of SOC level. The abscissa represents the number of adverse event reports, and the ordinate represents the adverse events of SOC level. **(B)** Case map of ADE of mycophenolate mofetil in the treatment of AIH at the PT level in FAERS.

At the PT level, notable signals included off-label use (95 cases; ROR = 2.80, 95%CI = 2.19–3.58; ROR, PRR, BCPNN) and drug ineffectiveness (45 cases; ROR = 3.34, 95%CI = 2.33–4.79; ROR, PRR, BCPNN). Additional significant PTs included product use in unapproved indication (27 cases; ROR = 2.01, 95%CI = 1.30–3.09; ROR, BCPNN), therapy non-responder (14 cases; ROR = 5.86, 95%CI = 2.88–11.94; ROR, PRR, BCPNN), and intentional product use issue (12 cases; ROR = 7.76, 95%CI = 3.41–17.63; ROR, PRR, BCPNN) (Supplementary Table S13; Figure 6B).

### 3.9 ADE signals of glucocorticoid-mycophenolate mofetil combination therapy in AIH

The analysis systematically identified significant ADE signals associated with the combination therapy of glucocorticoids and mycophenolate mofetil in AIH at the SOC level. Among the three significant SOC signals, eye disorders showed the highest ROR (4 cases; ROR = 2.88, 95% CI: 1.04–8.00; ROR, PRR), followed by respiratory, thoracic and mediastinal disorders (12 cases; ROR = 2.21, 95% CI: 1.21–4.05; ROR, PRR, BCPNN), and infections and infestations (25 cases; ROR = 1.57, 95% CI: 1.01–2.43; ROR) (Supplementary Table S14; Figure 7A). However, due to the small number of cases in the eye disorder signal, its robustness requires further evaluation.

**FIGURE 7:**
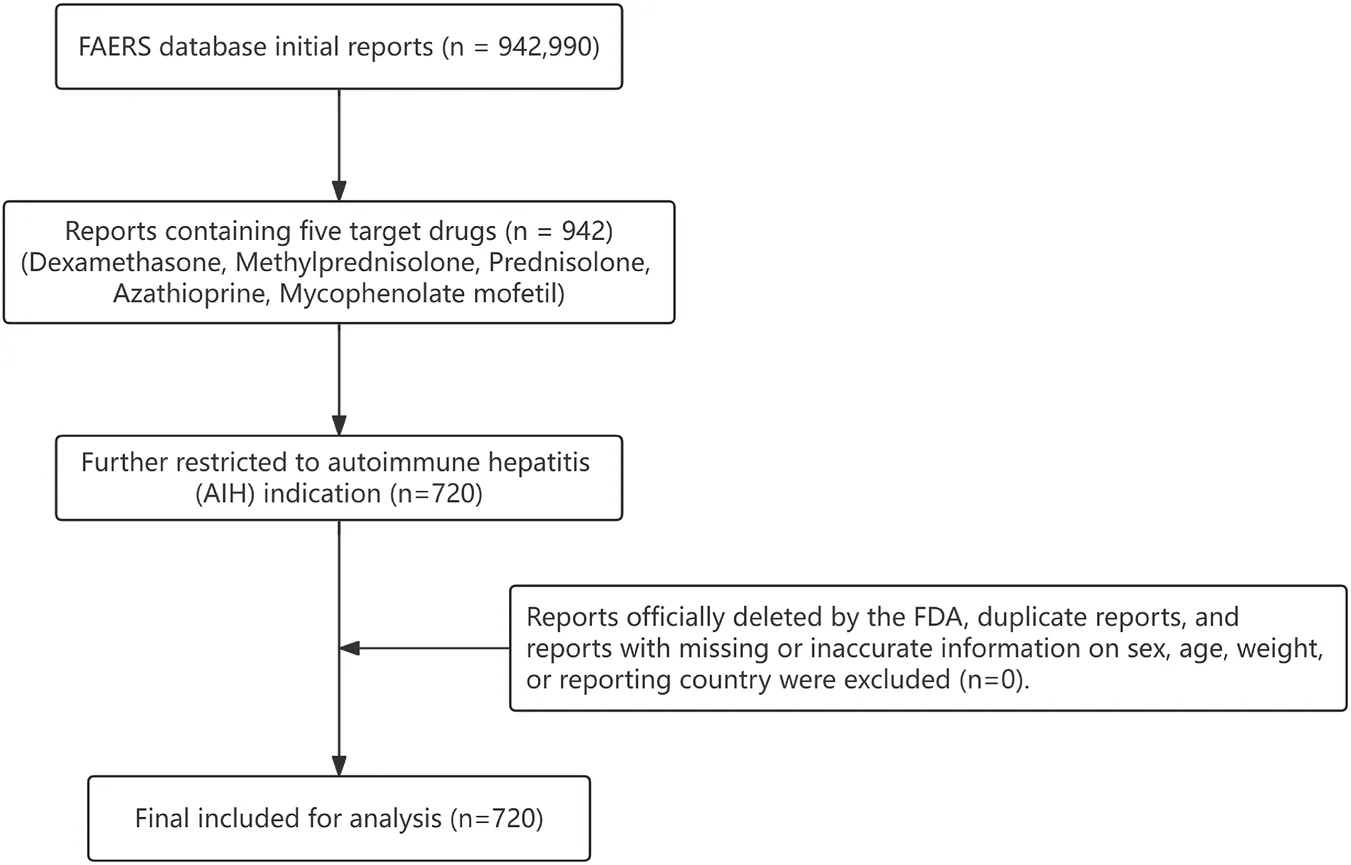
Significant ADE signals for glucocorticoid-mycophenolate mofetil combination therapy in AIH **(A)** Analysis of SOC level, with the abscissa being the number of adverse event reports and the ordinate being the adverse events of the SOC level **(B)** Case map of ADE of glucocorticoid combined with mycophenolate mofetil in the treatment of AIH at the PT level in FAERS.

At the PT level, the strongest signals identified were acute respiratory distress syndrome (4 cases; ROR = 49.42, 95% CI: 12.24–199.59; ROR, PRR, BCPNN), pathogen resistance (5 cases; ROR = 41.47, 95% CI: 12.51–137.46; ROR, PRR, BCPNN), *escherichia* sepsis (6 cases; ROR = 33.40, 95% CI: 11.73–95.10; ROR, PRR, BCPNN), acute respiratory failure (5 cases; ROR = 13.07, 95% CI: 4.81–35.50; ROR, PRR, BCPNN), and multiple organ dysfunction syndrome (4 cases; ROR = 8.96, 95% CI: 3.05–26.35; ROR, PRR, BCPNN) (Supplementary Table S15; Figure 7B). Of note, the limited number of reported cases for some PT-level signals may affect the precision of the association estimates; therefore, these signals should be interpreted with caution and warrant further validation in larger real-world datasets.

## 4 Discussion

AIH requires prolonged immunosuppressive therapy; however, the safety signal profiles of primary therapeutic agents and various treatment regimens remain incompletely characterized. This study employed the FAERS database to systematically analyze ADE reports associated with five principal and commonly utilized medications—dexamethasone, methylprednisolone, prednisolone, azathioprine, and mycophenolate mofetil—administered for AIH management from 2004 to 2024. This study utilized FAERS data updated to the fourth quarter of 2024 and used the descriptive statistics, disproportionality analysis, and stratified analysis to elucidate the specific safety signal profile of different therapeutic agents and regimens in the real-world AIH population. These findings provide evidence-based insights for optimizing AIH treatment strategies and enhancing adverse reaction surveillance in clinical practice.

Our demographic analyses of gender, age, and occupation profiles of adverse drug event (ADE) reports associated with AIH treatment highlight the need for targeted demographic consideration in clinical safety monitoring. In this study, 64.6% of eligible FAERS ADE reports were obtained from female patients, consistent with the well-recognized female predominance of AIH prevalence (Floreani et al., 2024). As such, the higher proportion of female cases in our dataset plausibly reflects the intrinsic demographic characteristics of the AIH patient population. Accumulated evidence has confirmed that sex-specific differences in drug-metabolizing enzymes (e.g., CYP3A4 and CYP2D6) and drug transporters (e.g., P-glycoprotein) can substantially affect systemic drug exposure and therapeutic responses (Poyonov et al., 2025; Aljohmani and Yildiz, 2026). However, due to the inherent limitations of FAERS as a spontaneous reporting system, whether these biological discrepancies directly contribute to the elevated ADE reporting frequency in female patients cannot be definitively determined. Future well-designed prospective pharmacokinetic and pharmacodynamic studies with rigorous control of confounding factors are therefore warranted to validate these mechanistic hypotheses.

The present analysis revealed significantly heightened adverse drug event (ADE) reporting signals for vascular and gastrointestinal disorders in patients aged ≥65 years. Advanced age is commonly accompanied by declined hepatic and renal function, which may impair drug metabolism and clearance and consequently result in systemic drug accumulation (Lindeman et al., 1985; Rzeczycki et al., 2025). Additionally, reduced gastric acid secretion and impaired gastrointestinal motility in elderly individuals may prolong mucosal exposure to therapeutic agents (Dumic et al., 2019; Stillhart et al., 2020). Furthermore, prevalent polypharmacy in older populations increases the likelihood of adverse events arising from drug–drug interactions (Chen et al., 2026). Collectively, these physiological and clinical characteristics may explain the stronger gastrointestinal ADE signals observed in elderly AIH patients in the current analysis. Notably, delayed diagnosis is common in elderly patients with AIH, and substantial impairment of hepatic functional reserve is often present at confirmation of diagnosis. Such clinical features render it challenging to differentiate gastrointestinal and systemic symptoms attributable to drug toxicity from those caused by disease progression, which may partially shape the age-specific distribution of ADE reporting frequency in older populations. It should be emphasized that several signals identified in the age-stratified analysis (such as vascular disorders) were derived from limited case numbers and thus represent exploratory findings. Moreover, the FAERS database lacks critical baseline information, including pre-existing organ function, concomitant medications, and exact drug dosages, precluding quantitative evaluation of the independent contributions of these confounding factors. Future large-scale cohort studies are warranted to validate these preliminary observations, with comprehensive adjustment for relevant covariates to clarify the independent modifying effect of age on the safety profile of immunosuppressive agents in AIH management.

An analysis of ADEs associated with glucocorticoid monotherapy in patients with AIH has identified primary safety signals centered on infections as well as metabolic and endocrine disorders. Notably, opportunistic infections, including *Candida*, Aspergillus, and Pneumocystis jirovecii pneumonia, along with indications of new-onset diabetes, emerged as particularly significant issues. These observations align with the established immunosuppressive mechanisms of glucocorticoids. For instance, research has demonstrated that glucocorticoids can reactivate latent pathogens, such as the Hepatitis B virus and *Mycobacterium tuberculosis*, by inhibiting T-cell function (Schweitzer et al., 2024). Furthermore, these agents markedly elevate the risk of invasive fungal infections, such as Aspergillus, in affected individuals (Farges et al., 2020). The immunosuppressive properties of glucocorticoids also contribute to an increased vulnerability to mucocutaneous candidiasis, including oral candidiasis and esophagitis (Reichel et al., 2024), as well as to bacterial infections caused by *Legionella pneumophila* and *Listeria* monocytogenes (Sivagnanam et al., 2017; Abad et al., 2019; Koopmans et al., 2023). Additionally, findings indicate that dexamethasone may induce insulin resistance, resulting in hyperglycemia (Oray et al., 2016). The opportunistic infection and metabolic signals identified in the present analysis are directionally consistent with the established safety profiles of these immunosuppressive agents, and the pharmacological mechanisms documented in previous literature provide plausible biological rationales for these observed associations. Nevertheless, considering the spontaneous nature of FAERS data, the correlations detected in this study remain statistical signals and cannot directly validate definite causal pathways. In addition, current clinical guidelines for AIH recommend against long-term monotherapy with glucocorticoids, given their well-documented cumulative toxic effects (Li et al., 2025). Nevertheless, short-term monotherapy is still occasionally administered in real-world clinical practice for patients who exhibit intolerance or contraindications to alternative immunosuppressive agents (Dyson et al., 2018). In such cases, our results help identify patients with higher reported ADE frequencies for targeted monitoring. In non-standard treatment settings, this study helps identify patients with prominent signals and guide focused monitoring. It shows that while combination therapies such as glucocorticoids with azathioprine may be associated with higher reporting of gastrointestinal issues, they are associated with lower reporting of infections compared to glucocorticoid alone. This suggests combination therapies could alter the distribution of reported ADEs and may be associated with lower reporting of glucocorticoid-related toxicities. The study’s analysis of glucocorticoid monotherapy signals aligns with current guidelines on known adverse effects and offers data for clinical monitoring decisions when such monotherapy is necessary.

Azathioprine (AZA), a widely utilized immunomodulatory agent in the management of inflammatory conditions, is frequently associated with a high incidence of adverse drug reactions (Casajús et al., 2022). A comprehensive analysis of adverse events reported in association with AZA monotherapy for AIH reveals that gastrointestinal disorders, dermatological conditions, and neoplasm-related signals are among the most frequently reported categories. Notably, while gastrointestinal disorders are the most commonly reported, resulting in treatment intolerance and drug discontinuation in approximately 5%–20% of patients (Zudeh et al., 2021), the analysis of association strength reveals that the reporting association with tumor development demonstrates the highest signal intensity. This finding underscores the significant potential for carcinogenicity associated with prolonged AZA use (Zelmat and Despas, 2024). Furthermore, hematological abnormalities, such as pancytopenia, along with adverse dermatological reactions, are frequently documented, emphasizing the multi-system toxicological profile of AZA (Casajús et al., 2022). Meanwhile, genetic factors also play an important role in the safety of AZA: for example, specific polymorphisms of the NUDT15 gene (such as rs116855232) have been confirmed to be important factors in predicting leukopenia caused by thiopurine drugs (Cargnin et al., 2018), and ITPA gene polymorphisms may also be associated with the risk of adverse reactions (Barba et al., 2022). Therefore, in clinical practice, for patients carrying the NUDT15 risk allele, the use of AZA should be avoided or initiated at extremely low doses with caution, accompanied by close monitoring (Chatterjee et al., 2025). In summary, this study systematically identifies safety signals of AZA in the treatment of AIH. Clinical monitoring and signal-based management should focus on related adverse drug reactions and incorporate genetic testing to achieve more targeted safety monitoring.

The analysis of ADEs associated with MMF monotherapy suggests a unique safety signal profile, primarily characterized by SOC signals related to injury, poisoning, and procedural complications, as well as PT-level signals represented by “treatment ineffectiveness”, “off-label use”, and “intentional product use issues”. This collectively indicates that MMF monotherapy may face challenges in medication management in clinical practice. MMF exhibits significant inter-individual variability in pharmacokinetics (Wei et al., 2022), and reports of treatment ineffectiveness in the database may be related to the significant pharmacokinetic variability of MMF. Fixed-dose regimens may lead to insufficient blood concentrations in some patients, thereby reducing efficacy (Mao et al., 2024). Nevertheless, spontaneous FAERS data cannot reliably delineate the potential association between documented treatment failure events and these pharmacokinetic variations. Further investigations incorporating therapeutic drug monitoring data are warranted to validate this underlying correlation. Notably, these signals also reflect, to some extent, the real-world context of MMF use in the AIH indication: the drug was initially developed as an anti-rejection agent for transplantation (Wang et al., 2022b), and the evidence base for its use in AIH is relatively limited, which may lead to off-label use or inconsistent efficacy expectations in clinical practice. Additionally, age-stratified analysis shows that elderly patients (≥65 years) have a significantly higher reporting frequencies for signals such as “intentional product use issues” and “death” highlighting the necessity for focused monitoring in this population. Although MMF demonstrates a superior safety profile compared to AZA in combination regimens (Dalekos et al., 2022; Ali et al., 2025), its monotherapy still faces issues of variable efficacy and the potential to be associated with fatigue and gastrointestinal disturbances, which may impact patients’ quality of life (Bhat et al., 2023). These findings suggest a distinct signal profile for MMF monotherapy in AIH management, indicating the necessity of focusing monitoring efforts on elderly patients and those with specific pharmacokinetic characteristics.

Analysis of combinational therapy with corticosteroids and AZA in patients with AIH revealed a significantly different adverse event profile compared to monotherapy. Among these, gastrointestinal adverse events were most common and were one of the main reasons for treatment discontinuation (Snijders et al., 2024), followed by infectious complications, which could manifest as septic shock in severe cases (Chastain et al., 2024). This aligns with the known signal profiles of the two drug classes: AZA is frequently associated with gastrointestinal intolerance, while the immunosuppressive effects of corticosteroids are associated with a higher reporting frequency of opportunistic infections. Although guidelines recommend tapering corticosteroids after disease remission to reduce risks (Pape et al., 2019), the signal analysis in this study suggests that this combination regimen still presents significant tolerability challenges. In contrast, while clinical trials indicate that MMF combined with prednisolone may achieve better biochemical responses, it may also be associated with a higher rate of adverse events and treatment discontinuation (Snijders et al., 2024), highlighting the differential signal profiles when different immunosuppressive agents are combined with corticosteroids. Therefore, for the combination therapy of glucocorticoids and AZA, the core of clinical management lies in the active monitoring of their established adverse effects, particularly those related to the gastrointestinal tract and infections.

The analysis of combination therapy with glucocorticoids and MMF highlights the dual aspects of prominent infectious signals and uncertainty regarding effectiveness. ADE analysis of glucocorticoid plus MMF combination therapy revealed prominent signals across multiple SOC and PT categories. Major SOC-level signals involved respiratory disorders, ocular diseases, and infections, while key PT-level events included acute respiratory distress syndrome, pathogen resistance, *Escherichia coli* sepsis, acute respiratory failure, and multiple organ dysfunction syndrome. Collectively, severe infectious and respiratory adverse events dominated the safety profile of this combination. MMF suppresses lymphocyte proliferation via purine synthesis inhibition, and its synergy with glucocorticoids amplifies immunosuppression, broadening infection spectra and elevating risks of opportunistic fungal and conditional bacterial infections (Chen et al., 2023; Shankar and Lamech, 2025). Despite biological plausibility, these observational findings are prone to indication and channeling biases, as combination therapy is typically prescribed for patients with more severe baseline disease. FAERS data cannot confirm causal mechanisms underlying these signals. The enriched respiratory signals in the combination group are consistent with prior pharmacovigilance studies linking immunosuppressant-glucocorticoid regimens to severe respiratory adverse events (Lai et al., 2025; Zhao et al., 2026). These consistent associations reflect known drug safety features but remain statistically suggestive, requiring prospective validation for causal inference. In summary, glucocorticoid-MMF combination therapy is primarily associated with severe infectious and respiratory adverse events. Clinical management requires intensified surveillance for opportunistic infections, drug-resistant pathogens, and progressive respiratory impairment. Considering the limited case volume of partial signals and the intrinsic limitations of spontaneous reporting systems in causal verification, large-scale prospective studies are warranted to validate the precise associations and clinical implications of these findings.

In managing AIH clinical safety, monitoring should target the specific signal profiles of each treatment option. For glucocorticoid monotherapy, focus on metabolic indicators and infection signs. AZA monotherapy requires pharmacogenetic screening, management of gastrointestinal symptoms, regular blood checks, and monitoring for tumor-related signals. MMF monotherapy should address efficacy uncertainties due to pharmacokinetic variability and enhance signal-based monitoring for elderly patients. For combination therapy with glucocorticoids and AZA, prioritize managing gastrointestinal intolerance and infection-related signals, with preventive measures and dynamic monitoring. The combination of glucocorticoids and MMF requires careful monitoring due to higher reporting frequencies of infection, uncertain efficacy from potential drug interactions, and metabolic toxicity like hyperglycemia. Intensive monitoring of infection signs, drug levels, and metabolic parameters is essential. Patient characteristics, such as age-related organ dysfunction, should inform monitoring plans but not directly alter drug choices without clear evidence. These recommendations enhance current guidelines to improve treatment safety based on real-world data. The findings of this study are largely congruent with key results from various prior analyses of the FAERS and clinical investigations, while also revealing several notable discrepancies. This study identified the preferred term (PT)-level “drug intolerance” signal across multiple treatment regimens. As a nonspecific, broad descriptor in the Medical Dictionary for Regulatory Activities (MedDRA), this term exhibits a low background reporting rate in FAERS, which may substantially inflate reporting odds ratio (ROR) values for targeted drug-exposed populations. This signal may therefore partly constitute artifacts arising from heterogeneous reporting behaviors and coding practices, requiring careful context-based clinical interpretation. Consistent with prior safety investigations of immunosuppressants, this signal supports that gastrointestinal intolerance is a leading cause of immunosuppressant discontinuation (39). Apart from drug intolerance, other detected signals were congruent with existing literature. In particular, the glucocorticoid-associated opportunistic infection signal aligns with established evidence that prolonged glucocorticoid exposure increases infection susceptibility (30). Furthermore, this study identified several rarely reported signals in previous AIH-related FAERS analyses, such as angina pectoris. Such discrepancies may stem from variations in data sources, enrolled population characteristics, and analytical methodologies across different studies, and the underlying causes remain to be further explored. Even so, the distinct signal profiles of different treatment regimens highlight the necessity of targeted clinical monitoring, while definitive monitoring priorities require validation through prospective investigations.

This study has several inherent limitations. As a spontaneous reporting system, FAERS is inevitably subject to underreporting, missing data and reporting bias, which prohibits causal inference and accurate estimation of true ADE incidence. Reports enrolled based on the “indication = AIH” field rely on voluntary reporter submission, which may cause case omission. Fully documented reports tend to represent severe or typical adverse events, introducing selection bias. Additionally, the absence of standardized data on drug dosage, treatment duration, hepatic reserve, cirrhosis stage, disease severity, concomitant medications, ethnicity and pregnancy status limits dose–response analysis, induction/maintenance phase stratification and in-depth subgroup analyses. Several signals with limited case numbers lacked multiple comparison correction, thereby carrying potential false-positive risks and only providing preliminary evidence. Critically, FAERS data are affected by indication confounding and channeling bias. Treatment regimens are prescribed according to individual patient characteristics, and baseline AIH severity can independently influence ADE occurrence, confounding the statistical association between drug exposure and adverse events. The lack of data on actual drug-exposed population size also precludes direct inter-drug safety comparisons and absolute risk calculation, such that detected signals cannot be simply attributed to drug toxicity. Methodologically, dexamethasone, methylprednisolone and prednisolone were pooled into a unified glucocorticoid group. Given their distinct potency and half-life differences, this integration may mask agent-specific safety signals. Missing dosage and treatment course data also hindered the differentiation of adverse events between induction and maintenance therapy. Furthermore, overlapping classification of combination therapy reports in corresponding monotherapy groups impedes the independent interpretation of individual drug safety profiles. To address these limitations, future prospective cohort and multicenter real-world studies are warranted. Studies with comprehensive covariates, well-defined exposed populations and standardized follow-up protocols will adopt multivariate adjustment and propensity score matching to control confounding factors, validating current findings in larger samples with external verification via other pharmacovigilance databases and clinical cohorts. Future work will also conduct separate safety assessments of individual glucocorticoids with dose stratification and treatment phase analysis to clarify scenario-specific risk profiles. Combined therapeutic drug monitoring and pharmacogenetic testing will be performed to evaluate the influence of genetic polymorphisms on individual drug responses. Ultimately, a multi-dimensional risk prediction model will be established to provide robust evidence for individualized immunosuppressive medication and targeted safety monitoring in AIH treatment.

## 5 Conclusion

This FAERS-based pharmacovigilance analysis revealed distinct adverse drug event profiles among key immunosuppressive therapies for autoimmune hepatitis. At the System Organ Class level, infections, gastrointestinal disorders, and neoplasms were the most frequently reported ADE signals, while drug intolerance was a prominent signal across multiple regimens at the Preferred Term level. Distinct safety signal patterns were identified among different AIH treatment regimens. Glucocorticoid monotherapy was associated with opportunistic infections and metabolic and endocrine abnormalities. Azathioprine monotherapy correlated with gastrointestinal intolerance and neoplasm-related adverse events. Mycophenolate mofetil monotherapy was linked to medication management issues and treatment inefficacy. Glucocorticoid combined with azathioprine primarily presented signals of gastrointestinal disorders, infections and drug intolerance, whereas glucocorticoid plus mycophenolate mofetil was predominantly associated with infections and severe respiratory adverse events. Female patients and older adults showed higher reported adverse event burdens, underscoring the importance of individualized surveillance. These findings provide real-world evidence to support more small case numbers precise safety monitoring and signal-based management in autoimmune hepatitis. However, because FAERS is a spontaneous reporting system, the observed associations should be interpreted cautiously and validated in large prospective studies.

## Data Availability

The original contributions presented in the study are included in the article/Supplementary Material, further inquiries can be directed to the corresponding authors.
