# Supplementary material for: Detection of adverse event signals associated with key immunosuppressants for autoimmune hepatitis: insights from the food and drug administration adverse event reporting system database
Source: Front Pharmacol. 2026 Sep 24;17:1889345. doi: 10.3389/fphar.2026.1889345 (PMC13645583; doi:10.3389/fphar.2026.1889345)
Supplement: Supplementary file 1 [file Table1.docx]

**Supplementary Table 1** Distribution of adverse event reports by treatment regimen among patients with autoimmune hepatitis (FAERS, 2004–2024)

| **Treatment regimen** | **Number of reports (n)** | **Drugs** | **Number of reports (n)** |
| --- | --- | --- | --- |
| Glucocorticoid monotherapy | 227 | Dexamethasone | 1 |
|  |  | Methylprednisolone | 58 |
|  |  | Prednisolone | 168 |
| Azathioprine (AZA) monotherapy | 193 |  |  |
| Mycophenolate mofetil (MMF) monotherapy | 300 |  |  |
| Glucocorticoid + AZA combination | 63 |  |  |
| Glucocorticoid + MMF combination | 39 |  |  |
