# Supplementary material for: Detection of adverse event signals associated with key immunosuppressants for autoimmune hepatitis: insights from the food and drug administration adverse event reporting system database
Source: Front Pharmacol. 2026 Sep 24;17:1889345. doi: 10.3389/fphar.2026.1889345 (PMC13645583; doi:10.3389/fphar.2026.1889345)
Supplement: Supplementary file 2 [file Table2.docx]

**Supplementary Table 2. A two-by-two contingency table and detailed formulas for disproportionality analysis**

|  | Target adverse drug event | Other adverse drug events | Sums |
| --- | --- | --- | --- |
| Target drug | a | b | a + b |
| Other drugs | c | d | c + d |
| Sums | a + c | b + d | a + b + c + d |

| Method | Formula | Threshold |
| --- | --- | --- |
| ROR | $ROR = \frac{a/c}{b/d}$ | $a\geq\left( lower limit \right)>1$ |
|  | $\mathrm{SE}\left( \mathrm{lnROR} \right)=\sqrt{\frac{1}{a}+\frac{1}{b}+\frac{1}{c}+\frac{1}{d}}$ |  |
|  | $95\%CI = e^{\ln\left( \mathrm{ROR} \right)\pm1.96SE\left( \mathrm{lnROR} \right)}$ |  |
| PRR | $PRR=\frac{a/\left( a+b \right)}{c/\left( \left( c+d \right) \right)}$ | $a\geq\left( lower limit \right)>1$ and $\chi^{2}>4$ |
|  | $\mathrm{SE}\left( \mathrm{lnPRR} \right)=\sqrt{\frac{1}{a}-\frac{1}{a+b}+\frac{1}{c}-\frac{1}{c+d}}$ |  |
|  | $95\%CI = e^{\ln\left( \mathrm{PRR} \right)\pm1.96SE\left( \mathrm{lnPRR} \right)}$ |  |
| BCPNN | $IC = \log_{2}\frac{p\left( x,y \right)}{p\left( x \right)p\left( y \right)} = \log_{2}\frac{a\left( a+b+c+d \right)}{\left( a+b \right)\left( a+c \right)}$ | IC025>0 |
|  | $E\left( \mathrm{IC} \right) = \log_{2}\frac{\left( a+1 \right)\left( a+b+c+d+\alpha\right)\left( a+b+c+d+\beta\right)}{\left( a+b+c+d+\gamma\right)\left( a+b+\alpha\right)\left( a+c+\beta\right)}$ |  |
|  | $V\left( \mathrm{IC} \right)=\frac{1}{\left( ln 2 \right)^{2}}\left[ \frac{\left( a+b+c+d \right)-a+\gamma-11}{\left( a+\gamma11 \right)\left( 1+a+b+c+d+\gamma\right)}+\frac{\left( a+b+c+d-\left( a+b \right)+a-\alpha1 \right)}{\left( a+b+\alpha1 \right)\left( 1+a+b+c+d+\alpha\right)}+\frac{\left( a+b+c+d+\alpha\right)-\left( a+c \right)+\beta-\beta1}{\left( a+b+\beta1 \right)\left( 1+a+b+c+d+\beta\right)} \right]$ |  |
|  | $\gamma= \gamma11\frac{\left( a+b+c+d+\alpha\right)\left( a+b+c+d+\beta\right)}{\left( a+b+\alpha1 \right)\left( a+c+\beta\right)}$ |  |
|  | $IC-2SD = E\left( \mathrm{IC} \right)-2\sqrt{V\left( \mathrm{IC} \right)}$ |  |
| MGPS | $\mathrm{EBGM} = \frac{a\left( a+b+c+d \right)}{\left( a+c \right)\left( a+b \right)}$ | EBGM05 > 2 and a > 0 |
|  | EBGM_05 =_ $e^{\ln\left( \mathrm{EBGM} \right)-2\sqrt{1.64(\frac{1}{a}+\frac{1}{b}+\frac{1}{c}+\frac{1}{d})}}$ |  |
